# Wood Ash as Sustainable Alternative Raw Material for the Production of Concrete—A Review

**DOI:** 10.3390/ma16072557

**Published:** 2023-03-23

**Authors:** Ece Ezgi Teker Ercan, Lale Andreas, Andrzej Cwirzen, Karin Habermehl-Cwirzen

**Affiliations:** 1 Building Materials, Department of Civil, Environmental and Natural Resources Engineering, Luleå University of Technology, 97187 Luleå, Sweden; andrzej.cwirzen@ltu.se (A.C.); karin.habermehl-cwirzen@ltu.se (K.H.-C.); 2 Waste Science and Technology, Department of Civil, Environmental and Natural Resources Engineering, Luleå University of Technology, 97187 Luleå, Sweden; lale.andreas@ltu.se

**Keywords:** wood ash, wood fly ash, forest waste, cement replacement, geopolymer, alkali-activated, concrete, mortar, supplementary cementitious materials (SCMs), ecological

## Abstract

Different ecological binders have been used to minimize the negative effects of cement production and use on the environment. Wood ash is one of these alternative binders, and there has been increasing research related to this topic recently. The wood ash utilized in the literature primarily originates from power plants and local bakeries, and predominantly wood fly ash is used. This review paper examines the use of wood ash as an ecological binder in two different applications: as a cement replacement and as an alkali-activated material. Studies have shown that while increased wood ash content in concrete and mortars can have negative effects on strength and durability, it is still a promising and developable material. Depending on the chemical composition of the wood ash, the strength and durability properties of concrete might be slightly improved by utilizing wood ash as a replacement for cement, with an optimal replacement level of 10–20%. However, there is a need for more research regarding the effects of wood ash on the durability of cement-based materials and its use in alkali-activated materials. Overall, this review provides a comprehensive overview of the properties of wood ash and its potential applications in conventional concrete and mortars, as well as in alkali-activated materials.

## 1. Introduction

Concrete is the most widely used material in the construction industry after water due to its good durability, mechanical properties, and low cost [1,2,3]. According to the Global Cement and Concrete Association, 14 billion m^3^ of concrete and 4.2 billion tons of cement were produced worldwide in 2020 [4]. Cement is the component of concrete that generates the largest carbon footprint; its production requires a significant amount of raw materials and energy, and the process results in the release of large amounts of CO_2_ into the atmosphere, contributing to environmental problems associated with greenhouse gas emissions. The predominant type of cement used in concrete production is Portland cement, which is primarily composed of clinker produced by burning limestone in cement plants. This process, known as calcination, decomposes CaCO_3_ into CaO and CO_2_, resulting in significant greenhouse gas emissions [5,6,7]. The global cement enterprise contributes about 5–8% of carbon dioxide emissions to the atmosphere [8,9]. Depending on the source of energy, the emitted amount of CO_2_ during cement production is estimated to be between 500 and 900 kg CO_2_/t cement [10].

To address the environmental effects related to cement manufacturing, for instance, the depletion of natural resources, the choice of supplementary cementitious material to secure sustainable concrete needs to be strengthened. There is also a growing global demand for carbon-efficient solutions that reduce CO_2_ emissions and utilize waste. This trend is consistent with the goals of the Paris agreement, which requires urgent action to prevent the global temperature increase from exceeding 1.5 °C [11]. Over the years, fly ash, which is an industrial by-product, has been used to replace cement and produce more durable and economical construction materials. However, the source availability of fly ash is diminishing because coal-based thermal power plants are being closed worldwide [12]. Biomass and wood ash are sustainable alternatives to fly ash [13,14].

Wood ash consists of organic and inorganic residues formed as a result of burning wood and wood products. When wood is burned, an average of 6–10% of its weight is turned into ash [15]. Globally, the production of woody biomass is approximately 4600 million tons per year, out of which 60% is used for energy production, 20% is used for industrial purposes, and the remaining 20% is the primary production loss that decomposes in the field [16]. Today, a significant proportion of wood ash is disposed of in landfills, while some is utilized in the domains of agriculture and forestry [17,18]. Nevertheless, some concerns exist regarding these applications of wood ash. Firstly, it is predicted that costs will increase due to difficulties in finding a landfill area in the future [7,19]. In addition, landfilling of wood ash may cause the leaching of hazardous elements and may cause contamination of groundwater [20]. The disposal of wood ash through landfilling may give rise to concerns regarding potential health risks, as fine particles can become airborne and be dispersed by wind [21,22]. The utilization of some agricultural practices may pose potential risks due to the presence of heavy metal content and acidic pH levels [23]. In comparison to other wood ash disposal methods, the use of wood ash in concrete might represent a more sustainable alternative [15].

Quality control of wood ash is more difficult than that of coal fly ash, which is widely used in concrete production. This is because the mixture of the organic and inorganic content of wood ash may differ regarding various factors such as species and parts of the tree, geographical location of the growing tree, combustion technology and temperature, collection method from the boiler, and storage conditions [6,18,24,25,26]. Although the use of conventional fly ash in concrete is a common practice, the use of wood ash in concrete is not yet in accordance with EN 450-1 [27] and ASTM C618 [28].

**Figure 1:**
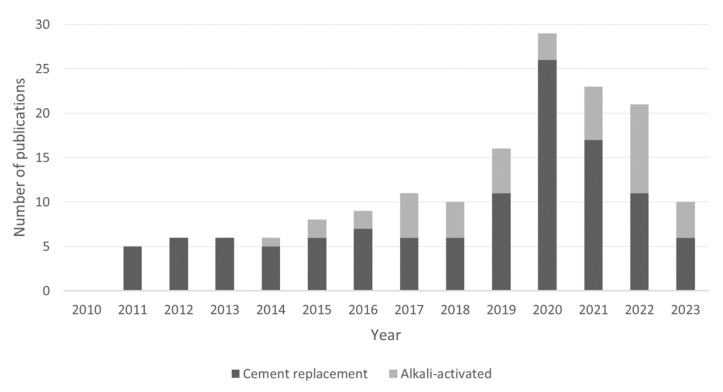
Number of publications listed in Scopus related to wood ash utilization in construction materials between 2010 and 2023 (accessed on 9 March 2023).

Although wood ash does not yet meet the usage recommendations in the standards, previous scientific studies show that it can be used in the construction industry [20,21,24,29,30,31,32]. The number of scientific publications published on the use of wood ash in these two applications between 2010 and 2023 is given in Figure 1. The paper reviews the understanding of the use of wood ash in concrete with two different applications, namely partial cement replacement in traditional mortars and concretes and in the use of alkali-activated materials.

## 2. Characteristics of Wood Ash

### 2.1. Physical Properties

**Figure 2:**
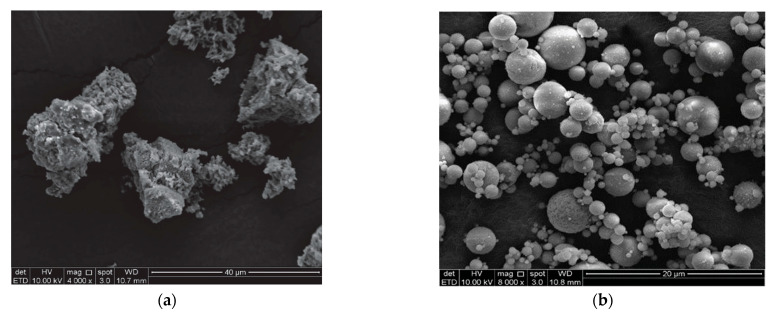
FESEM images of (**a**) wood ash and (**b**) fly ash (reprinted from Abdulkareem et al. [38], with permission of AIP Publishing).

**Figure 3:**
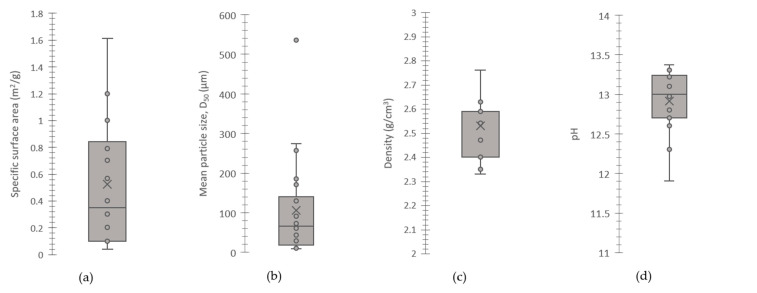
(**a**) The specific surface area [21,39,40], (**b**) mean particle size [21,24,39,41,42], (**c**) density [17,24,30,42], and (**d**) pH [21,42] values of various wood ashes reported in the literature.

Surface area and particle size are important parameters for setting and water demand [33]. A finer particle size affects the reactivity positively [32]. Berra et al. [24] conducted experiments on three different types of wood ash. The particle size ranges were between 86 and 176 µm. Moreover, the real density that they found ranged between 2.35 g/cm^3^ and 2.76 g/cm^3^. Since the density of wood ash is significantly lower than that of cement, there is a high reduction in unit weight when it is used as a cement replacement. Rajamma et al. [30] compared the bulk density and specific surface areas of two different fly ashes generated from forest residues (F1) and the pulp and paper industry (F2). F1 had a density of 2.59 g/cm^3^ and a specific surface area of 40.29 m^2^/g, while F2 had a density of 2.54 g/cm^3^ and a specific surface area of 7.92 m^2^/g. The high difference between the surface areas is attributed to the irregular particle shape and the high amount of unburnt organic matter of F1. Carević et al. [32] stated that wood bottom ash particles are coarser than wood fly ash and cement particles. The particle sizes of the wood bottom and fly ash ranged between 10 and 1000 µm and between 0.2 and 100 µm, respectively. Wood bottom ash had spherical particles with irregular morphology. Many authors observed that wood ash particles have a porous structure [6,24,30,32]. Wood ash has larger and more irregular particles and larger specific surface areas compared to Portland cement [30,34,35,36,37]. Moreover, Abdulkareem et al. [38] observed wood fly ash particles have higher surface porosity and are more angular than fly ash particles (Figure 2). The ranges of the specific surface area, mean particle size density, and pH values of various wood ashes reported in the literature are given in Figure 3.

### 2.2. Chemical Properties

The binder characteristic depends on components such as CaO, SiO_2_, Al_2_O_3_, and Fe_2_O_3_. According to EN 450-1 [27], the chemical composition of fly ash for use in concrete meets the following requirements by mass: the sum of pozzolanic oxides, which are SiO_2_, Al_2_O_3_, and Fe_2_O_3_, should be higher than 70%; reactive SiO_2_ higher than 25%; reactive CaO lower than 10%; the total of alkali content, which is Na_2_O and K_2_O, lower than 5%; MgO lower than 4%; chloride (Cl^−^) content lower than 0.10%; SO_3_ less than 3%. Etiégni and Campbell [18] stated that wood ash is a highly alkaline material (pH = 9–13.5) due to its carbonate, bicarbonate, and hydroxide content. High P_2_O_5_ content might cause a delay in the setting of the concrete. High levels of alkali in wood ash might have a negative impact on concrete durability due to the occurrence of alkali–aggregate reactions. Similarly, high levels of SO_3_ might cause sulfate attack, which can result in the deterioration of the concrete structure [21,32,42]. Sklivaniti et al. [43] observed hydroxide and carbonate content which gives high alkalinity with a pH value of 11.7. Wood ash has higher amounts of LOI, CaO, K_2_O, P_2_O_5,_ and MgO than coal fly ash [44,45]. Ukrainczyk et al. [6] reported that the main mineral phases of the ashes are lime (free CaO), MgO, larnite (2CaO·SiO_2_), and calcium carbonate (CaCO_3_) [6]. Etiégni and Campbell [18] compared the content of ashes produced at combustion temperatures between 538 and 1093 °C. They observed Ca, K, Mg, Si, and P as the major components, and the general trend was an increasing metal content with increased combustion temperature. However, K, Na, and Zn contents decreased when the temperature was increasing. They attributed this to the low decomposition points of carbonates and oxides. Berra et al. [24] studied three types of wood fly ash obtained from the combustion of chestnut, poplar virgin wood chips, and the production of scraps of treated wood. The washing treatment was used for the wood ashes in order to reduce the content of sulfates, alkalis, and chlorides. Sigvardsen et al. [46] also used washing treatment on wood ash and observed that washing treatment removed the soluble compounds. Siddique [15] stated that the density of wood ash decreases with increasing carbon content. Vassilev et al. [47] compared 28 types of wood ash and observed significant differences in the oxide contents due to the type of wood, combustion, transportation, and storage of the ashes. Moreover, they determined the decreasing order of the mean of the oxide content as CaO > SiO_2_ > K_2_O > MgO > Al_2_O_3_ > P_2_O_5_. The chemical components of different types of wood ashes obtained by using different combustion temperatures and methods in the literature are given in Table 1.

### 2.3. Mineralogical Analysis

Etiégni and Campbell [18] observed lime, calcite, portlandite, and calcium silicate as major components. Elinwa and Mahmood [48] found silicates and carbonates to dominate in the studied sawdust ash. Ngueyep et al. [55] stated that wood ash can be used as a cement replacement due to its content of amorphous silica. In addition to the amorphous phase, calcite, gypsum, anhydrite, quartz, tridymite, magnetite, hematite, rutile, and muscovite were observed as crystalline phases. Rajamma et al. [30] examined the results of XRD analysis of wood ash-containing cement pastes to observe the effect of wood ash on the formed phase. They stated that in addition to the main peaks of calcium hydroxide, calcium aluminum hydrate, and calcium silicate, they also observed ettringite, calcite, and silica peaks. They observed that the calcium silicate peaks were more intense in the samples containing cement and 10% wood ash compared to pastes containing 30% wood ash. Furthermore, the ettringite formation was attributed to the increasing alkali and water content with increasing wood ash content. Chowdhury et al. [41] also observed that wood ash contained SiO_2_ in both amorphous and crystalline forms.

### 2.4. Loss on Ignition

Loss on ignition (LOI) shows the unburnt organic content in the ash which is a result of an uncontrolled or incomplete incineration, and it might vary according to the analysis temperature [29,30,32]. The standard testing methods ASTM C311 [56] and EN 196-2 [57] prescribe different temperatures for the measurement of LOI: 750 ± 50 °C and 950 ± 25 °C, respectively. The maximum allowable limits for LOI in ASTM C618 [28] vary depending on the type of fly ash, falling within the range of 6–10%, while EN 450-1 [27] specifies limits between 5 and 9%. Despite these differences in the standard methods, many studies in the literature do not specify the temperature at which LOI is measured, and in some cases, the stated measurement temperatures were between 750 and 1000 °C. According to ASTM C618 [28], if acceptable performance results are provided, the use of Class F fly ash with loss on ignition of up to 12% is acceptable. Ngueyep et al. [55] state that if the LOI value is greater than 12%, the pozzolanic activity reduces due to the unburnt carbon, and wood ash acts as a filler in the concrete mixture. The high amount of unburnt carbon in the wood ash might significantly affect the pozzolanic and durability properties, workability, setting, and mechanical strength [24,32,58]. High LOI values might also have an impact on the effectiveness of chemical admixtures [32]. It might delay hydration [24] and create problems in making air-entrained concrete [12,59]. Sklivaniti et al. [43] found the LOI of wood bottom ash was 42% and attributed this to the fact that carbonation during combustion led to the formation of CaCO_3_ and K_2_Ca(CO_3_)_2_. Carević et al. [42] stated that high LOI is also a result of the decomposition of hydrated and carbonated wood ash phases.

Various methods have been proposed to reduce the unburnt carbon content in the ash, which is an important issue in the utilization of wood ash as a supplementary cementitious material in concrete. Doudart de la Grée et al. [60] suggested using a 500 µm sieve to decrease the LOI through the removal of large carbon particles of the ash. Amaral et al. [39] used a re-calcining treatment on wood ash, which was shown to decrease the LOI from 24.30% to 10.60%. In addition to reducing loss on ignition, pre-treatment methods such as grinding and water-washing treatments might also have positive effects on the physical and chemical characteristics of wood ash which might influence the strength and durability properties of concrete [24]. Grinding can increase particle reactivity by reducing the particle size [60], whereas water-washing treatments can remove the chloride, alkali, and sulfate content [24].

### 2.5. Pozzolanic and Hydraulic Properties

Pozzolanic activity is defined as a reaction between calcium hydroxide and alumina silicates, resulting in a hydration product with binding properties [61]. In EN 450-1 [27], the pozzolanic property is determined by the sum of the amounts of SiO_2_, Fe_2_O_3,_ and Al_2_O_3_, also known as pozzolanic oxides, and this sum must be greater than 70%. According to the literature, the sum of pozzolanic oxides in wood ashes varies in the range of 13.03% [53] to 88.32% [8]. Many studies have reported that wood ash shows pozzolanic behavior [30,41,48,62]. Elinwa and Mahmood [48] reported pozzolanic properties due to the sum of pozzolanic oxide of sawdust ash being 73.55%. Rajamma et al. [30] also observed pozzolanic activity using the Frattini test for wood fly ash, although the pozzolanic oxide content was 53.2%. Ramos et al. [8] determined the pozzolanic properties of wood ash by replacing 20 wt% of Portland cement and determining the strength activity index according to EN 450-1 [27].

On the other hand, some studies have reported no pozzolanic property. For example, Garcia and Sousa-Coutinho [31] assessed the pozzolanic activity of different wood fly and bottom ashes using the Frattini test, which showed no pozzolanic activity. Demis et al. [63] attributed the lack of pozzolanic property to the low amount of SiO_2_ (31.8%) and high LOI (27%).

Materials that form hydration products as a result of chemical reactions with water have the ability to harden and maintain their strength and stability even under water after hardening and are defined as hydraulic binders. As a result of the reaction of cement with water, the C-S-H gel is formed as the main hydration product. Portlandite, which increases the high alkalinity of the solution and thus helps to protect the reinforced concrete from corrosion, is also formed during hydration [21,53,64]. The hydraulic activity mainly depends on the SiO_2_ and CaO content. As specified in EN 197-1 [65], the ratio of CaO to SiO_2_ should be greater than 2. Cheah and Ramli [66] stated that due to the high CaO content of wood ash, it might also show hydraulic behavior. Berra et al. [24] did not detect any pozzolanic activity according to EN 196-5 [67]. The observed compressive strength increase was explained by the hydraulic property [24].

## 3. Properties of Concrete and Mortar Containing Wood Ash

### 3.1. Workability

There is a consensus that workability decreases with increasing ash ratio in blended cement [6,20,24,58,68,69]. Berra et al. [24] identified that the reduction in workability of concrete containing wood fly ash is attributed to the porous wood fly ash particles’ irregular shape and higher specific surface area, which differ from those of Portland cement particles. Moreover, the workability of the concrete was observed to decrease as loss on ignition (LOI) of wood fly ash increased. However, the authors also noted that the workability was improved by a water-washing treatment. Similarly, Ukrainczyk et al. [6] agreed that an increase in the wood replacement ratio tends to lower the workability of concrete, which may be linked to the larger particle size of wood ash compared to Portland cement. The workability can be enhanced by the addition of a superplasticizer. Carević et al. [68] compared the workability of samples with 5, 10, and 15 wt% cement replacement by woody biomass ash. A decrease in workability was observed with increasing wood ash ratio, which the authors related to the porous particle microstructure of wood ash. It was also indicated that the woody biomass ash could accelerate the hydration process, leading to higher temperatures and faster loss of workability. On the contrary, Carević et al. [42] stated that the decrease in workability cannot be a result of the temperature increase since the temperature increase difference between the reference sample and the mortar with 15 wt% wood ash content was only 3%. Moreover, Hamid and Rafiq [58] observed a shear slump due to low workability. Rajamma et al. [34] stated that the replacement of cement by 10 wt% of wood ash did not influence the workability of mortars. Brazão Farinha et al. [70] also reported that a cement replacement of up to 15 wt% of wood ash did not affect the workability. On the other hand, Yang et al. [71] observed a slight increase in workability in samples containing 10 wt% of wood ash. However, they agreed that the 20 wt% and 30 wt% replacement ratios generally tend to decrease the workability. This was related to the high amount of unburnt carbon in the wood ash absorbing water, which reduced the free water content in the fresh mixture. The slump values reported in the literature and the effects of wood ash on the different properties of concrete and mortar are represented in Table 2.

### 3.2. Setting Time

Many studies have shown that the incorporation of wood ash generally leads to delayed setting time of a system based on Portland cement. Moreover, this delay increases with the increase in the replacement ratio [24,29,42,49,58,71]. Carević et al. [42] reported a delay in setting and attributed this to high alkali and magnesium oxide contents. Chen et al. [72] stated that the use of fly ash with a high LOI value in concrete may delay the setting time. They also pointed out that this phenomenon can be improved by adding setting accelerator additives or by increasing the curing temperature. Yang et al. [71] also observed a longer setting time for 20 and 30 wt% wood ash-containing samples that was attributed to the C_3_A concentration. On the other hand, they also observed the setting time of samples containing low amounts of wood ash (e.g., 10 wt%) was shorter than that for the control sample with 100 wt% Portland cement. The high CaO and alkali contents of wood ash were indicated as the main reasons. When 10 wt% wood ash was used, the alkalis in the wood ash helped to dissolve the aluminate and silicate ions of the cement, and the setting was accelerated. However, in the case of using 20 wt% and 30 wt% wood ash, the alkali concentration increased and slowed the setting by preventing Ca^2+^ dissolution. Sklivaniti et al. [43] also reported shorter setting times with increasing wood ash percentages. This was attributed to the mineralogy of the used wood ash, the formation of carboaluminates, and carbonates present during the cement hydration. In addition, it has been stated that the ash can reduce the setting time by worsening the workability of the cement paste due to its small particle size. Rajamma et al. [30] also observed a shorter setting time with increasing wood ash content in samples containing 20 wt% and 30 wt% of wood ash. This has been related to increased water consumption by the present organic matter.

### 3.3. Soundness

The stability of the volume change during the setting and hardening processes is defined as soundness [73]. Elinwa and Mahmood [48] observed that the soundness of test samples increased with the increase in sawdust ash amount. Carević et al. [42] characterized six different wood ashes and observed that only one of them had free CaO content within the limit standards specified in EN 450-1 [27] (<1.5% for free CaO). However, according to soundness test results, all tested cement paste mixes that were replaced with 5, 10, or 15 wt% of wood ash met the criteria of the EN 450-1 [27] standard.

### 3.4. Compressive Strength

Generally, the compressive strength decreased with an increasing wood ash ratio, but with some exceptions. Elinwa and Ejeh [49] compared the compressive strengths of sawdust waste fly ash additive samples with different cement replacement ratios, i.e., 0, 5, 10, 15, 20, and 30 wt%. Samples were cured for 3, 7, 28, and 60 days. After 60 days of curing, the average compressive strength value of reference samples was 22.44 MPa. The highest compressive strength value of the wood ash-containing samples was reached for samples containing 10 wt% replacement and equaled 21.45 MPa. It was concluded that the compressive strength decreased with an increasing ash ratio. Udoeyo et al. [20] used sawdust and wood-shaving ashes with 0, 5, 10, 15, 20, and 30 wt% replacement ratios. After 90 days, the recorded compressive strengths were as follows: for reference, 31.48 MPa; for 5 wt%, 28.66 MPa; for 10 wt%, 27.54 MPa; and 19.52 MPa when 30 wt% replacement was used.

On the other hand, Rajamma et al. [30] observed an increase in the 28-day compressive strength but only for concretes containing 10 wt% of wood ash. Garcia and Sousa-Coutinho [31] used ground wood bottom ash in mortars with 5 wt% and 10 wt% cement replacement. After 3 months, the compressive strength improved by 12% in comparison with reference mixes. Subramaniam et al. [74] produced concrete blocks using 0, 10, 15, 20, and 25 wt% replacements of cement with wood ash. The best-measured result was 3.66 MPa and was obtained after 21 days for the mix containing 15 wt% of wood ash. The compressive strength of concrete blocks without wood ash additive was 3.10 MPa. The 15-day compressive strength of samples with 25 wt% of wood ash decreased after 14 days. Carević et al. [42] indicated that increasing wood ash ratio, free CaO, and alkali content were the main factors for the decrease at an early age strength.

### 3.5. Split Tensile Strength

The split tensile strength showed similar trends to those described for the compressive strength [17,50,75]. Naik et al. [75] reported the split tensile strengths for reference concrete and the concrete mixes containing 0, 5, 8, and 12 wt% of wood ash; the split tensile strengths were 3.8 and 4.3 MPa for reference samples and 3.6–4.0 MPa and 4.2–5.1 MPa for the samples containing wood ash [17]. The optimum replacement for the split tensile strength was reported as 8 wt% [75,76]. Chowdhury et al. [41] stated that the tensile strength decreases with increasing wood ash replacement levels at a slower rate than observed for compressive strength. It was related to poor bonding between wood ash and mortar matrix. Lessard et al. [77] explained the reduction in the split tensile strength by the higher porosity of matrixes incorporating wood ash. Udoeyo and Dashibil [50] reported that the split tensile strength of the samples with sawdust ash at 7 and 28 days was reduced with an increasing cement replacement ratio. A more significant reduction was observed after 7 days. However, up to a 25 wt% replacement ratio, the split tensile strength reached 90% of the 28-day strength of the control sample. Akinyemi and Dai [78] attributed the decreasing tensile strength with increasing ash content to increased flocculation, which reduces the ability of the system to resist tensile forces.

On the other hand, Raju et al. [79] reported 14.3, 12.1, 10.8, 11.5, and 6.2% improvements in the split tensile strength for concrete samples containing 5, 10, 15, 20, and 25 wt% of the wood ash, respectively. Amaral et al. [39] used wood ash that was either calcinated or ground as 15 and 30 wt% of Portland cement. The results showed that the water/binder ratio and the amount of used wood ash have the strongest effects on the splitting strength. The tensile strength of samples having the w/b ratio of 0.35 was independent of the cement replacement ratio and the used pre-treatment methods. At higher w/b ratios, i.e., 0.50 and 0.65, the tensile strength values were almost the same as or slightly higher than the reference sample value. Exceptions were samples containing 30 wt% of the ground and calcined wood ash. They also reported an existing correlation between compressive strength and tensile strength.

### 3.6. Flexural Strength

Generally, the incorporation of wood ash tends to reduce flexural strength [8,17,20,30,80]. However, Garcia and Sousa-Coutinho [31] observed that at later ages, it can be improved. Udoeyo et al. [20] tested the flexural strength of samples containing wood ash with different replacement ratios (5, 10, 15, 20, 25, and 30 wt%). The measured 28-day flexural strength tended to decrease with increasing wood ash content. The flexural strength of concretes containing 5 wt% wood ash reached 5.20 N/mm^2^ and 3.74 N/mm^2^ when 30 wt% replacement was used. Rajamma et al. [30] tested the flexural strength of the samples containing 10 wt%, 20 wt%, and 30 wt% of wood ash. The flexural strength decreased with increasing wood ash amount. For wood ashes with high silica content, it is recommended to use a maximum replacement ratio of 20 wt% to keep the flexural strength at an acceptable level [76]. Garcia and Sousa-Coutinho [31] stated that the flexural strength of mortars with 5 wt% and 10 wt% wood ash replacement showed a similar trend to compressive strength but at a slower rate for 3 months. However, at the end of 90 days, the mortar containing 5 wt% wood ash and the control sample have the same flexural strength values. After 365 days, an improvement of 18% was observed in the flexural strength of the mortars containing 5 wt% wood ash when compared to the control sample, and an improvement of 11% was observed in those containing 10 wt% wood ash. Chowdhury et al. [41] also observed that flexural strength decreased with increasing wood ash content regardless of the water-to-binder ratio. The authors noted that the reduced strength properties with increasing ash content could result from poor bonding with the matrix.

### 3.7. Water Absorption

Generally, water absorption increases with the increase in wood ash ratio [24,29,58]. Studies have shown that water absorption in concrete tends to increase as the wood ash ratio in the mix is increased [19,25,45]. Udoeyo et al. [20] investigated the effect of waste wood ash replacement on the water absorption of cement, testing samples with varying ash replacement ratios (5–30 wt%). They observed that as the ash replacement ratio increased, the water absorption ratio also increased. The water absorption ratios for samples containing 5, 15, and 30 wt% of ash were 0.4%, 0.8%, and 1.05%, respectively. It is generally acceptable for construction materials to have a water absorption ratio of less than 10 wt%. The water absorption capacity of mixes containing wood ash was found to increase with the water-to-cement ratio as well [39]. On the other hand, Elinwa and Ejeh [49] reported a reduction in water absorption with the addition of wood ash. In their study, 50 mm cube samples were prepared using 15 wt% sawdust ash, and water absorption tests were conducted after 28 days of curing. The water absorption ratios of the control sample and the samples containing 15 wt% sawdust ash were measured at 1.29% and 0.8%, respectively.

### 3.8. Shrinkage

Low drying shrinkage is important as it can reduce the formation of microcracks in concrete [22,75]. Naik et al. [75] reported that wood ash significantly reduced the drying shrinkage of concrete [22]. Cheah and Ramli [66] studied the shrinkage behavior of mortars containing high-calcium wood ash as a partial cement replacement. The results showed that 5 wt% wood ash significantly reduced the drying shrinkage, in both early and later ages. This reduction was attributed mostly to the autogenous shrinkage due to the dilution of cement content. Although the shrinkage of samples containing 10 wt% of wood ash was measured slightly higher than that of the reference sample at an early age, more reduction was observed at a later age. However, at higher replacement ratios (15, 20, and 25 wt%), both in early and late ages, the shrinkage of mortars was higher than that of the reference sample. Carević et al. [42] measured the drying shrinkage of pastes and mortars containing different amounts (5, 10, and 15 wt%) of wood ash after 1, 3, 7, 14, 28, and 58 days, and after that, it was measured every month for 1 year. They obtained the wood ashes from different combustion technologies, namely a grated combustor, pulverized fuel combustor, and bubbling fluidized bed combustor. Maximum combustion temperature might change according to combustion technology. It can be 900 °C in a fluidized bed combustor, 1000–1200 °C in a grate combustor, and around 1600 °C in a pulverized fuel combustor [37,42,81]. Usually, at temperatures higher than 900 °C, free CaO is formed, and it might affect the long-term properties negatively [42,82]. They observed that the incorporation of wood ash reduced the drying shrinkage regardless of the replacement ratio and combustion technology. On the other hand, Candamano et al. [80] reported that the use of wood ash increased drying shrinkage and weight loss from an early age. The highest drying shrinkage was measured at a 30 wt% cement replacement ratio. The contribution of autogenous shrinkage to the total shrinkage decreases with a decreasing cement amount and an increasing amount of wood ash. The increasing shrinkage due to the presence of the wood ash was related to the hygrometric shrinkage, which was confirmed by the increased weight loss.

### 3.9. Frost Durability

In cold regions, concrete is subjected to repeated freezing and thawing cycles. During freezing, the water expands, causing internal stress in the concrete which may lead to microcracking and scaling [83]. In addition, at low temperatures, concrete strength development decreases by 20–40% because the rate of hydration is reduced [84]. High LOI might have a negative effect on the air-entraining admixtures for concrete that otherwise has a good resistance to freezing and thawing [27]. It is specified that the incorporation of wood ash with 5, 8, and 12 wt% of cement replacement does not significantly affect the frost resistance of concrete [17,75]. Wang et al. [85] examined the freezing and thawing behavior of concrete containing wood fly ash (WFA), fly ash (Class F and C), the mix of wood fly ash (20 wt%) and Class F fly ash (80 wt%) (Wood F), and the mix of wood fly ash (20 wt%) and Class C fly ash (80 wt%) (Wood C). The fly ash/cement ratio was 25 wt% for all mixes. The weight loss of samples containing Class C fly ash was either equal to or lower than that of the reference sample. The presence of wood ash appeared to have a low impact on frost durability. The authors also agreed that wood ash has a low impact on freezing and thawing behavior. The air-entraining agent requirement of the mixtures is listed as Class F > Wood F > Wood C > Class C > pure Portland cement.

### 3.10. Chloride Permeability

High permeability accelerates ion and moisture transfer within the concrete and can cause chemical erosion or attacks. Rapid chloride permeability mainly depends on the water/binder ratio, curing conditions, and age. Wang et al. [85] compared the rapid chloride permeability of wood fly ash and coal fly ash as partial cement replacements. The results showed a high chloride permeability of samples containing the wood ash. The large particle size was indicated as the main factor. Garcia and Sousa-Coutinho [31] also reported that 5 and 10 wt% wood ash-containing samples had slightly higher chloride permeability than the control sample. However, they were all categorized as having a low level of resistance to chloride permeability [31,86].

### 3.11. Alkali–Silica Reaction

Alkali–silica reaction (ASR) occurs between reactive silica present in concrete aggregates and alkalis from the pore solution. As a result, an alkali–silica gel is formed around the reactive aggregates. This gel tends to expand when a sufficient amount of water is provided. The tensile stresses might develop and result in expansion leading to the cracking of the binder matrix [87,88]. High alkali content and LOI might cause substantial alkali–silica reaction (ASR) expansion risks [85].

Some test results indicate that the presence of wood ash could mitigate the alkali–silica reaction and expansion [8,89,90]. For example, Esteves et al. [89] investigated the properties of wood ash that had been sieved (75 μm) and milled, as well as washed to remove soluble salts. They prepared mortar samples that contained 20 and 30 wt% of wood ash with a highly reactive fine aggregate. Other mixes also contained 20 wt% of wood ash and 10 wt% of metakaolin. The results showed a reduction in the alkali–silica reaction expansion with an increasing amount of wood ash. Moreover, metakaolin reduced the expansion significantly. Ramos et al. [8] also studied mortars containing 10 and 20 wt% of wood ash as cement replacement with reactive fine aggregate. The alkali–silica reaction expansion was reduced by about 27% and 72% for 10 and 20 wt% replacements, respectively. Wang and Baxter [90] highlighted that although biomass fly ash contains more alkali than Class C coal fly ash, the reduction in expansion was greater. Moreover, the expansion was stopped at 0.1% after 6 months with biomass fly ash-containing mixes, while Class C fly ash significantly exceeded the limit specified in the ASTM C33 [91]. This phenomenon was related to the higher alkali content of wood ash.

## 4. The Use of Wood Ash in Alkali-Activated Materials

Alkali-activated cementitious binders are based on a reaction of an aluminosilicate precursor with alkalis. The main reaction product of an alkali-activated system with high calcium is calcium(alumino)silicate hydrate gel [92,93,94]. Alkali-activated materials have been identified as a potential alternative to traditional Portland cement-based materials due to their reduced carbon footprint, higher strength, and superior durability properties [92,95,96]. However, they typically require the addition of alkali activators, such as sodium silicate and sodium hydroxide, which may have negative environmental impacts [95,97]. Additionally, the curing process for some alkali-activated materials typically requires elevated temperatures, which can increase production costs [98]. Wood ash, which is a promising replacement material for cement, offers an additional advantage as it contains high levels of alkali that can be utilized as an alkali activator [98]. In addition, wood ash can be used as a replacement material for granulated blast furnace slag, fly ash, and metakaolin in alkali-activated materials. The literature findings on alkali-activated wood ash are summarized in Table 3.

Bajare et al. [99] conducted a study to compare the effect of two different curing temperatures on the compressive strength and water absorption of geopolymer mortars containing wood ash that had been ground using a planetary ball mill to improve reactivity. The curing temperatures used in the study were 20 °C and 75 °C, and 6M sodium hydroxide was used as the alkali activator. The results showed that the compressive strength of the mortar samples cured at 75 °C for 24 h was 9.3 MPa, while the samples cured at 20 °C had a compressive strength of 2.3 MPa. In addition, the authors observed microcracking on the samples cured at 75 °C, which they attributed to the rapid reaction process or thermal shock. The water absorption of the samples also decreased with increasing curing temperature.

Silva et al. [92] used wood ash as a precursor and NaOH solution with different concentrations (2 M, 3 M, 4 M, 5 M) as an alkali activator. In addition, glass powder with different replacement ratios was used in some of the mixes. The compressive and flexural strength values of mixes containing 100 wt% of wood ash varied in the ranges of 2.85–3.69 MPa and 0.64–0.83 MPa, respectively. The highest compressive strength values were reached for the 2M NaOH concentration. High porosity and some unreacted wood ash particles were observed in the matrix of the samples containing 100 wt% wood ash. No C-(A)-S-H gel was observed in any of the studied samples. Wood ash replacement by glass powder with 30, 35, and 40% improved compressive and flexural strength to values of 7.20–19.62 MPa and 2.45–4.41 MPa. The strength tended to increase with the increase in NaOH concentration and the decrease in glass powder ratio. Samples containing glass powder had higher density and lower amounts of microcracks compared to the reference samples. At the same time, the microstructure was porous, cracked, and heterogeneous, containing unreacted and partially reacted particles. The formed microcracks were attributed to the drying process during curing. Lower strength values were attributed to the presence of coarser particles. Consequently, sieving and crushing processes were suggested as pre-treatments for wood ash and glass powder.

Abdulkareem et al. [96] studied the effect of the partial replacement of fly ash with wood ash in geopolymer mortars at replacement ratios of 10, 20, and 30 wt%. The results showed that the inclusion of wood ash reduced the initial and final setting times of the blended mixtures. The compressive strength of the samples with 10–20 wt% wood ash at early ages (3–7 days) was improved due to the early formation of geopolymer gels and C-S-H minerals. The inclusion of wood ash at up to 20 wt% resulted in low water absorption and total porosity at early ages, but increased water absorption at later ages (28 days).

Cheah et al. [100] replaced fly ash with 50, 60, 70, and 100 wt% of high-calcium wood ash in geopolymer mortars prepared without any alkali activator. Wood ash contains high amounts of K_2_O and CaO that are suitable for the geopolymerization process. When it reacts with water, the wood ash forms K-A-S-H and C-A-S-H gels, as well as C-S-H gel. The study found that the maximum compressive strength of 18 MPa was achieved in the sample with 60 wt% wood ash after 90 days of curing.

Cheah et al. [40] replaced fly ash with high-calcium wood ash with 10, 20, 30, 40, 50, 60, 70, 80, 90, and 100 wt% replacement ratios in geopolymer mortar. The wood ash was sieved with a 600 µm sieve to remove carbon and large particles. The specific gravity and specific surface were 2.43 and 5671 cm^2^/g, respectively. A sodium silicate solution with an alkali modulus of 2.1 was used as an alkali activator. The highest compressive and flexural strength was achieved with 40 and 50 wt% wood ash-containing samples at 7 and 28 days. However, they also observed that the 30 wt% wood ash-containing samples had the highest mechanical strength after 365 days. As the wood ash content in the binder increased, the amount of mixing water required to achieve standard consistency also increased.

Owaid et al. [101] also used wood ash as fly ash replacement with 25, 50, 75, and 100 wt% ratios in geopolymer concrete. The alkali solution used was a mix of sodium silicate and sodium hydroxide, with a ratio of 2.5 (SS/SH) and 10 M concentration. The specimens were cured at 60 °C for 24 h, followed by room-temperature curing. The results revealed that the GC mix containing 25 wt% wood ash exhibited the highest compressive strength on the 56th day, measuring 57.82 MPa. However, mixes with higher percentages of wood ash replacement resulted in a decrease in the compressive strength, splitting tensile strength, and flexural strength when compared to the reference sample. This decrease in strength was attributed to the high CaO content present in wood ash [102,103]. The workability of the geopolymer mixes was found to not be significantly affected by the incorporation of wood ash. The study suggests that partial replacement of fly ash with wood ash up to 25 wt% can be economically and environmentally feasible for use in GC production.

Samsudin and Cheah [52] investigated the use of high-calcium wood ash as a replacement material for ground granulated blast furnace slag (GGBS) in geopolymer concrete. The study utilized replacement ratios of 0, 10, 20, 30, 40, 50, 60, 70, 80, 90, and 100 wt% and was carried out without the use of a chemical alkali activator. Results of the study indicated that the optimal replacement ratio was 30 wt%, which yielded the highest compressive strength at 12.3 MPa. Furthermore, the study highlighted that the highest rate of compressive strength development occurred during the early stages of curing, which is believed to be attributed to the high alkalinity of wood ash that promotes the dissolution of aluminosilicate materials, leading to rapid compressive strength development. Additionally, the research findings also revealed an increase in water demand as the wood ash content increased.

Cheah et al. [98] found that the inclusion of fly ash in blended geopolymer mortars made with high-calcium wood ash, granulated blast furnace slag (GBFS), and a low amount of alkali activator improved the mechanical and microstructural properties. The mortars were prepared with GBFS and wood ash in a ratio of 80:20 and replaced with fly ash at levels of 0, 10, 20, 30, 40, 50, 60, 70, 80, 90, and 100% by weight. The addition of fly ash at levels of 10–60% decreased the water demand and increased both the initial and final setting times. The initial and final setting times of the reference sample (80 wt% GBFS and 20 wt% wood ash) were measured as 20 and 90 min, respectively. The authors attributed this fast setting to the absorptive property of wood ash and the high calcium content. The maximum compressive strength of 49.61 MPa at 90 days was achieved with the sample containing 80 wt% fly ash. The improvement in mechanical and microstructural characteristics at fly ash replacement levels of up to 60 wt% was attributed to the formation of C-A-S-H and N-A-S-H gels at early ages and prolonged aging. However, samples with 100 wt% fly ash did not develop any compressive strength due to the low alkali activator content used in the preparation process.

Candamano et al. [104] investigated the effect of incorporating wood ash into geopolymer mortars as a partial replacement for metakaolin on the workability and mechanical properties of the resulting mortars. They found that the incorporation of 10, 20, and 30 wt% wood ash improved workability and resulted in a more porous structure with unreacted wood ash particles. However, the compressive and flexural strength of the mortars decreased with wood ash replacement levels above 10 wt%. Despite this decrease in strength, the mortars still exhibited a compressive strength of over 35 MPa even with 30 wt% wood ash replacement. The inclusion of wood ash also resulted in a slight increase in both drying shrinkage and weight loss, likely due to the increased porosity.

De Rossi et al. [105] examined the influence of various curing conditions on the properties of geopolymer mortars made from 75 wt% wood fly ash and 25 wt% metakaolin. They used two different ratios of sodium silicate to sodium hydroxide (SS/SH = 1 and 1.5) and subjected the samples to five different curing methods: thermal curing (TC) at 40 °C for 28 days in a hot chamber, hydrothermal curing (HC) at 40 °C for 28 days in hermetic bottle, submerged curing (SC) at room temperature and humidity for 1 day and then in 20 °C water for 27 days, room curing (RC) at 20 °C and 65% humidity for 28 days, and usual curing (UC) at 40 °C for 1 day and 20 °C and 65% humidity for 27 days. The results showed that the higher SS/SH ratio of 1.5 improved the compressive strength of the geopolymers compared to the lower ratio of 1, likely due to the higher SiO_2_/Al_2_O_3_ molar ratio in the material. Geopolymers made with a lower concentration of sodium silicate (SS/SH = 1) had higher water absorption than those made with a higher concentration (SS/SH = 1.5) unless they were cured using hydrothermal methods. The highest compressive strength was observed in geopolymers made with SS/SH = 1.5 and cured using thermal methods (24.2 MPa), while those cured using hydrothermal methods had the lowest strength (18.3 MPa). While thermal curing resulted in the highest compressive strength, it was not recommended due to its high energy consumption and carbon dioxide emissions. The researchers found that curing under room conditions (RC) was a cost-effective and environmentally friendly method for producing geopolymers with equivalent or improved mechanical properties compared to the other methods tested.

**Table 3:** Alkali activators, curing methods, or temperature of alkali-activated materials with wood ash.

Materials and Precursor Replacement Ratios	Alkali Activator	Curing Method/Temperature	Findings	References
Mortars with 100 wt% wood ash	SH (6M)	20 °C and 75 °C (for 24 h) then 20 °C	-Heat curing improved compressive strength (9.3 MPa).	[99]
Mortars with 0, 10, 20, 30, 40, 50, 60, 70, 80, 90, and 100 wt% of GGBS replacement with high-calcium wood ash	-	ambient temperature	-The optimum replacement ratio was determined as 30 wt% with the highest compressive strength (12.3 MPa). -Increasing wood ash content increased the water demand.	[52]
Mortars with 50, 60, 70, 80, 90, and 100 wt% of fly ash replacement with high-calcium wood ash	-	ambient temperature	-Acceptable strength and durability properties were observed without any alkali activator and elevated temperature curing.	[100]
Mortars with 0, 10, 20, 30, 40, 50, 60, 70, 80, 90, and 100 wt% of fly ash replacement with high-calcium wood ash	SS (Ms = 2.1)	ambient temperature for 24 h and then samples were wrapped to prevent moisture.	-The optimum wood ash content was determined as 30 wt% after 365 days.	[40]
Mortars with 0, 10, 20, 30, 40, 50, 60, 70, 80, 90, and 100 wt% of fly ash replacement with the mix of GGBS (80 wt%) and wood ash (20 wt%)	SS + SH	ambient temperature	-Wood ash has absorptive property due to the high calcium content and shortened setting time.	[98]
Mortars with 0, 10, 20, and 30 wt% of metakaolin replacement with wood ash	SS + SH	70 °C for 1h and then the ambient temperature	-Compressive and flexural strength decreased for a higher replacement ratio than 10 wt%. -Incorporation of wood ash slightly increased drying shrinkage, weight loss, and porosity.	[80]
Mortars with 0, 10, 20, and 30 wt% of fly ash replacement with wood ash	SS + SH	70 °C for 24 h and then room temperature	-Compressive strength improved up to 20 wt%. -Initial and final setting times decreased.	[96]
Mortars with 75 wt% wood ash and 25 wt% metakaolin	SS + SH (SS/SH = 1 and 1.5)	-40 °C for 28 days in a hot chamber (TC) -40 °C for 28 days in a hermetic bottle (HC) -at room temperature and humidity for 1 day and then in 20 °C water for 27 days (SC) -20 °C and 65% humidity for 28 days (RC) -40 °C for 1 day and 20 °C and 65% humidity for 27 days (UC)	-RC was reported as a cost-effective and environmentally friendly method. -Samples with a higher SS/SH ratio showed more improvement in compressive strength. -Higher water absorption was observed in the samples with SS/SH = 1 (except HC curing).	[105]
Mortars with 100 wt% wood ash and 30, 35, and 40 wt% of wood ash replacement with glass powder (GP)	SH (2, 3, 4, and 5M)	room temperature	-For 100 wt% wood ash, 2 M NaOH-containing samples had the highest compressive strength. -GP incorporation improved the compressive strength. However, it tended to increase with the increase in NaOH concentration and decrease in GP ratio.	[92]
Concrete with 25, 50, 75, and 100 wt% of fly ash replacement with wood ash	SS + SH (SS/SH = 2.5)	60 °C for 24 h and then at room temperature	-Compressive strength decreased with increasing wood ash ratio. The highest compressive strength was 57.82 MPa for 25 wt% wood ash-containing samples on the 56th day. -Similar workability was observed in all mixes.	[101]

SS: sodium silicate (Na₂SiO₃), SH: sodium hydroxide (NaOH).

## 5. Sustainability of Wood Ash

Incorporating wood ash into concrete has the potential to yield significant environmental benefits, particularly considering the increasing costs associated with landfilling [25,39,106]. This approach aligns with the principles of the circular economy, which advocates for sustainable development and efficient resource utilization with the goal of achieving zero carbon emissions [107]. The circular economy aims to use the end-of-life of products as an economic resource and emphasizes the responsible use of resources and sustainable consumption [108,109]. To promote sustainable development and efficient waste management, the construction industry is increasingly adopting circular economy principles, focusing on reducing waste and maximizing the reuse, recycling, and recovery of resources [109,110]. However, to effectively utilize wood ash as a valuable resource in contributing to a more sustainable urban infrastructure, it is necessary to develop innovative and ecologically acceptable recycling strategies that are environmentally conscious [39,51]. This approach ultimately promotes an environmentally conscious and socially responsible future.

Wood biomass is highly regarded as a source of energy due to its CO_2_-neutrality, as it emits almost the same amount of CO_2_ when burnt as it absorbs during its growth [39,42,111]. Replacing cement with wood fly ash has been found to have significant environmental benefits in the construction industry. Life cycle analysis has been used to evaluate the impact on humans and the environment, and opportunities for improvement have been identified [5,112]. Teixeira et al. [5] compared the life cycle assessments of different types of fly ashes and found that all types of fly ash reduced environmental impacts by decreasing cement consumption and CO_2_ emissions. Biomass fly ash had the best environmental performance when used as a replacement for cement. Gaudreault et al. [113] found that the most environmentally promising applications for wood ash are its use as an agricultural land amendment, a forest soil amendment, and a partial replacement for Portland cement according to the life cycle assessments. The authors recommended that the wood ash disposal method should be determined according to the chemical characteristics of wood ash that is locally available in the market.

Wood ash can be used in various construction materials, such as self-compacting concrete, lightweight foamed concrete, and restoration mortar for historical buildings as hydraulic lime [37,114,115,116,117]. Moreover, it can be used in brick and panel production due to its low density, reducing thermal conductivity and increasing heat capacity. It can be considered a sustainable insulation alternative that reduces energy losses in buildings [118,119,120].

Abdulkareem et al. [121] assessed the feasibility of using conventional and alternative precursors in geopolymer mixes in terms of the three main pillars of sustainability: environmental, economic, and social aspects. Local availability of materials is considered a social indicator. Using locally available materials results in lower transportation emissions, increased domestic supply security, and decreased dependence on imports. Conventional precursors such as fly ash, blast furnace slag, and metakaolin might have lower energy consumption and environmental impact, as they require less pre-treatment. However, alternative precursors may require additional pre-treatment such as grinding and sieving to achieve the desired properties, resulting in increased energy consumption and costs [121]. The average energy consumption for grinding and sieving is 11.0 kW h/t and 2.2 kW h/t, respectively [122]. Amaral et al. [39] compared grinding and re-calcinating as pre-treatment methods and found that recalcination was not eco-efficient due to energy consumption and did not improve mechanical or durability properties. Therefore, selecting the appropriate pre-treatment method is crucial for ensuring sustainability.

Kannan et al. [123] conducted a comparative analysis of the costs associated with high-strength concretes containing wood ash and metakaolin. The study found that the use of 40 wt% wood ash and 40 wt% metakaolin as replacements for cement resulted in cost reductions of 32.77% and 6.56%, respectively, compared to concrete containing only cement. Moreover, the highest compressive strength was achieved in concrete samples containing a mixture of 15 wt% wood ash and 25 wt% metakaolin, which resulted in a cost reduction of 16.51%.

## 6. Conclusions

The study showed that wood ash can be categorized as a sustainable material that can be used in the production of ecological concretes. The following conclusions were formulated:

- The chemical composition and quality of wood ash depend on many factors such as raw material origin, production, and storage parameters. It is important to characterize the raw material very frequently.
- The chemical composition is highly variable; usually, it contains high amounts of CaO and SiO_2_ and has a high loss on ignition.
- Wood ash has larger, porous, and irregular particles and a larger specific surface than Portland cement. For this reason, the workability tends to decrease as the wood ash content increases.
- Pre-treatment methods such as sieving, washing, and grinding have a positive effect on the fresh mix workability.
- The setting time is usually delayed with an increasing wood ash amount, with some exceptions.
- The use of wood ash as a partial cement replacement results in slightly worse or better mechanical properties in comparison with conventional Portland cement-based concrete.
- The replacement ratio is the dominant factor, and similar trends were observed for compressive, flexural, and split tensile strengths. In general, an increase in the proportion of wood ash resulted in a slight reduction in these properties. The optimum wood ash level is 10–20 wt% for cement replacement.
- Water absorption usually increased with increasing wood ash content.
- Incorporation of wood ash slightly improved shrinkage. In addition, it might mitigate the alkali–silica reaction expansion.
- Wood ashes do not have a significant effect on frost durability.
- The use of wood ash increased the chloride permeability.
- The use of wood ash in alkali-activated materials has yielded results with variations depending on the type and concentration of alkali activator, curing conditions, and the presence of other binders in the mix. While an increase in wood ash content generally resulted in a reduction in mechanical properties, some improvements were observed at levels up to 30 wt%. Moreover, wood ash can be used as an alkali activator.
- Wood ash is a promising sustainable material in terms of environmental, economic, and social aspects. Nonetheless, its local availability and the identification of suitable pre-treatment methods are important factors for mitigating environmental impacts and reducing cost.

The utilization of wood ash as a supplementary cementitious material in ecological concretes has gained increasing attention as a potential means of reducing the environmental impact of cement production. Despite the promising results in the literature, there is not enough research on the effects of wood ash on the durability properties of cement-based and alkali-activated materials, and the underlying mechanisms are not fully understood. Furthermore, while pre-treatment methods have demonstrated potential in improving the physical and chemical properties of wood ash, further investigation is needed to fully explore the extent of their efficacy.

## Figures and Tables

**Table 1:** Chemical composition and loss on ignition of different wood ashes (wt%).

Ash Type	Combustion		SiO_2_	Al_2_O_3_	Fe_2_O_3_	Sum of Pozzolanic Oxides	CaO	MgO	K_2_O	Na_2_O	TiO_2_	P_2_O_2_	SO_3_	Cl^−^	MnO	LOI	References
	Method	Temperature (°C)															
Sawdust	Open burning	-	67.20	4.09	2.26	73.52	9.98	5.80	-	0.08	-	0.48	0.45	-	-	4.67 ^a^	[48,49]
Sawdust	Open burning	-	78.92	0.89	0.85	80.66	0.58	0.96	-	0.43	-	-	-	17.93	-	8.40 ^a^	[50]
Wood	Local bakery oven	-	31.8	28	2.34	62.14	10.53	9.32	10.38	6.5	-	-	-	-	-	27 ^a^	[29]
Wood	-	-	73.01	11.93	3.38	88.32	2.64	1.03	4.14	3.81	0.48	0.59	<0.05	0.009	-	1.47 ^a^	[8]
Wood	GF	1000	41	9.30	2.6	52.9	11.4	2.30	3.9	0.9	0.4	0.3	-	-	0.3	25 ^b^	[30,34]
Wood	GF	-	15	2.59	3.98	21.57	55.50	2.66	10.7	0.64	0.51	0.9	1.4	-	-	-	[6]
Wood	-	-	47	8.70	5.10	60.8	17.40	3.3	6.7	1.0	-	3.1	3.0	0.6		5.1 ^a^	[51]
Wood	-	800	2.70	1.30	1.30	5.30	61.0	8.70	12.0	-	0.11	2.70	2.80	0.10	0.86	18 ^a^	[38,52]
Wood	GF	800	39.95	10.50	4.23	54.68	16.25	4.30	4.77	1.32	1.17	1.35	0.60	-	-	8.3 ^a^	[53]
Wood	GF	500–1000	19.8	6.16	2.85	28.81	46.75	8.26	6.05	0.64	0.34	1.82	2.73	-	-	3.8 ^a^	
Wood	PF	700–750	9.28	2.28	1.47	13.03	51.90	3.75	9.20	0.54	0.15	1.84	3.58	-	-	13.8 ^a^	
Wood	GF	600–1000	11.0	2.4	2.9	16.3	53.6	4.2	14.6	1.0	-	2.9	5.4	0.8	-	15.0 ^c^	[46]
Wood *	GF	600–1000	12.7	3.0	3.2	18.9	65.0	5.8	4.4	1.0	-	3.8	1.3	0	-	19.6 ^c^	
Wood	CFB	760–930	23.8	5.6	3.1	32.5	44.7	4.1	7.6	0.8	-	3.8	6.1	0.4	-	16.2 ^c^	
Wood *	CFB	760–930	26.5	6.3	3.3	36.1	45.0	4.4	5.7	0.9	-	4.2	3.7	0	-	19.7 ^c^	
Wood	GF	600–1000	8.6	1.9	2.3	12.9	48.9	3.8	16.8	2.2	-	-	5.4	-	-	15 ^c^	[54]
Wood	CFB	760–930	21.8	4.9	2.7	29.4	45.2	4.0	7.2	0.8	-	-	5.8	-	-	16 ^c^	

GF, grate firing combustion; BFB, bubbling fluidized bed combustion; CFB, circulated fluidized bed combustion; PF, pulverized fuel combustion. * Washed wood ash. ^a^ Temperature for LOI is not reported. ^b^ LOI at 1000 °C. ^c^ LOI at 950 °C.

**Table 2:** Effects of wood ash incorporation on mortars and concretes.

Material and Cement Replacement Ratio	Properties	Results	Observed Effects of Wood Ash	References
Mortar 0, 5, 10, 15, 20, 25, and 30 wt%	Setting time Soundness Compressive strength	116–190 min (initial), 241–337 min (final) 0.70–1.45 mm 3.70–22.44 MPa (at 3–60 days)	-Pozzolanic activity -Increased water demand, setting time, and soundness -Optimum wood ash ratio: 10 wt%	[49]
Concrete 0, 10, 20, 30, and 40 wt%	Setting time Slump Compressive strength	100–436 min (initial), 160–789 min (final) 30–40 mm (w/b = 0.60, 0.66, 0.67, 0.68, 0.69) 8.59–24.15 MPa (at 28–60 days)	-Pozzolanic activity -Increased water demand and setting time -Optimum wood ash ratio: 20 wt%	[29]
Concrete 0, 5, 10, 15, 20, 25, and 30 wt%	Slump Compressive strength Flexural strength Water absorption	0–8 mm 12.83–28.66 MPa (at 3–90 days) 3.51–5.20 MPa (at 3–90 days) 0.14–1.05%	-Decreased workability (up to 20 wt%), and slump was not observed for higher levels -Optimum wood ash ratio: 5 and 10 wt%	[20]
Mortar 0, 10, 20, and 30 wt%	Slump Setting time Compressive strength Flexural strength	110–130 mm 120–150 min 22.59–43.31 MPa (on the 28th day) 3.39–6.98 MPa (on the 28th day)	-Pozzolanic activity -Acceptable strength results up to 20 wt% wood ash	[30]
Mortar 0, 10, and 20 wt%	Strength Activity Index Alkali–silica reaction (ASR) expansion	98–102% (at 28–90 days) 0.1643% for 10 wt% wood ash, 0.00669% for 20 wt% wood ash at 14 days	-Pozzolanic activity -Increased compressive strength -Decreased ASR expansion with increasing wood ash content	[8]
Mortar 0, 5, and 10 wt%	Compressive strength Flexural strength Resistance against chloride permeability	32.2–65.4 MPa (at 3–365 days) 8–11.5 MPa (at 3–365 days) Low (on the 78th day)	-No pozzolanic activity -Decreased strength with increasing wood ash content -12% improvement in compressive strength after 3 days -Slightly higher chloride permeability	[31]

## Data Availability

No new data were created or analyzed in this study. Data sharing is not applicable to this article.
